# Periodontal diseases and all-cause dementia risk: Genetic instrument analyses in half a million UK Biobank participants

**DOI:** 10.1177/13872877261450558

**Published:** 2026-05-21

**Authors:** Chenyi Gao, Mark M. Iles, David Bunce, Bei Wu, Huabin Luo, Susan Pavitt, Jianhua Wu, David Timothy Bishop, Jing Kang

**Affiliations:** 1 School of Dentistry, 4468 University of Leeds, Leeds, UK; 2 105714 Wolfson Institute of Population Health, Queen Mary, University of London, London, UK; 3 Leeds Institute for Data Analytics, 4468 University of Leeds, Leeds, UK; 4 NIHR Leeds Biomedical Research Centre, Leeds Teaching Hospitals NHS Trust, Leeds, UK; 5 School of Psychology, 4468 University of Leeds, Leeds, UK; 6 Rory Meyers College of Nursing, 15935 New York University, New York, NY, USA; 7 Department of Public Health, 3627 East Carolina University, Greenville, NC, USA; 8 Leeds Institute of Medical Research, School of Medicine, 4468 University of Leeds, Leeds, UK; 9 Centre for Oral Clinical Translational Sciences, Faculty of Dentistry, Oral and Craniofacial Sciences, 4616 King's College London, London, UK

**Keywords:** aging, Alzheimer's disease, casual inference, dementia, genetics, gum diseases, neurodegeneration diseases, periodontal diseases

## Abstract

**Background:**

Numerous studies suggest that periodontal diseases might be associated with the development of dementia, but the causality is inconclusive.

**Objective:**

This study aims to explore the casual effect of periodontal diseases on all-cause dementia.

**Methods:**

The UK Biobank (UKB) data (n = ∼500,000) has been implemented, where participants were divided into two independent groups (2/3train and 1/3test). The exposure is the self-reported periodontal diseases, and the outcome is all-cause dementia measured by both clinical diagnoses based on ICD10 and ICD9 codes, and self-reported dementia. Four sets of genetic instruments were developed based on four different thresholds (main approach: p < 5 × 10^−8^; alternative approach I: p < 5 × 10^−6^; alternative approach II: p < 10^−4^; and alternative approach III: the best-fit p-value threshold calculated by polygenic risk score). The causal association between periodontal diseases and dementia was assessed by inverse-variance weighted (IVW), MR-Egger regression, weighted median, and mode-based estimate models.

**Results:**

The number of genetic instruments included in these four approaches varied from 3 to 1020, after passing the MR assumption checks. Most MR results suggested no causal association between periodontal diseases and dementia except the IVW model from main approach (coefficient beta: −0.816, 95% confidence interval, CI (−1.617, −0.015)) and the weighted median model from alternative approach II (beta: 0.077 95%CI (0.006, 0.149)) suggested potential causal relationship between periodontal diseases and dementia.

**Conclusions:**

The results showed inconsistent evidence of causal link between periodontal diseases and dementia using UKB. Future studies are needed with clinically defined periodontal diseases to better understand the causal link.

## Introduction

Periodontal diseases, especially periodontitis (severe periodontal disease), are potentially risk factors for dementia, as suggested by many observational studies, both longitudinal and cross-sectional.^1–3^ However, with limited direct biomedical evidence, common confounders (e.g., smoking, nutrition intake), and possible reserve causation, it is still unknown whether periodontal diseases can be casually associated with dementia.

Mendelian randomization (MR), using genetic variants as instruments, is a popular tool nowadays to assess the causal association between exposure and disease outcome. The advantage of MR is stated elsewhere,^4^ but the biggest challenge for MR approach is to find the relevant and reliable genetic instruments that is truly associated with exposure and not the disease outcome. Our previous study systematically reviewed the genetic variants of periodontitis from all high-quality genome-wide association studies (GWAS) of periodontitis, and not a single common genetic variant was found from the 15 included studies, indicating the heterogeneity of the genetic instruments in periodontitis.^5^ To date, there are two MR studies investigated causation between periodontitis and cognitive impairments or Alzheimer's disease using public databases (the FinnGen database and the GLIDE consortium), but neither found evidence of a causal link and no consistent genetic instruments of periodontitis were selected across these two studies by using the publicly available summary statistics.^6,7^ Selecting genetic variants in the MR approach should be cautious to avoid potential false positives, and applying various thresholds and approach like Polygenic Risk Score (PRS) is needed.^8^

UK Biobank (UKB, https://www.ukbiobank.ac.uk/) is high quality and ideal resource to investigate, as it contains information of participants’ genetic variants, oral health condition, and clinically diagnosed or self-reported dementia cases. Our previous study used UKB data and genome-wide association approach and identified four possible genetic loci associated with periodontal diseases, and still none was consistent with previous literature.^9^ In this study, we aim to investigate the causal association between periodontal diseases and all-cause dementia using four different selection criteria for the genetic instruments with standard MR models (IVW, MR-Egger regression, weighted median, and mode-based estimate). Given the fact that genetic instruments for periodontal diseases from previous studies or public databases were not reliable nor consistent, we divided the UKB participants into two independent groups, as training set and testing set, to independently identify genetic instruments of periodontal diseases using four different approaches.

## Methods

### Participants and data resource

This study used individual level of UKB data and included only the European participants (n = 409,548) from the final release of UKB genetic data (UKB project number 54633). The European ancestry was determined by self-reported ethnicities and its similarity with genetic ancestry computed by principal components analysis.^10^ Participants who registered withdrawal (n = 139) at the time of analysis were excluded from this study.

### Study design

Two-sample MR approach was applied because we divided UKB participants into two independent groups: a periodontal diseases group and a dementia group (details in ‘sample allocation’ section). Various genetic instruments selection methods were applied to understand such causal association comprehensively, including setting up different cut-off level of p-values and PRS approach for the best-fit p-value threshold genetic instruments selection, which is less biased due to weak instruments.^8,11^

### Diseases definition

Periodontal diseases were measured by self-reported dental health questions from UKB (variable code: 100538). Participants who have reported “painful gum, bleeding gum or loose tooth” were classified as ‘periodontal diseases cases’, while participants who reported other oral conditions were classified as ‘not having periodontal diseases’. The self-reported measurement of periodontal diseases is valid and a reliable surrogate for periodontal diseases,^12,13^ and the same UKB periodontal diseases definition has been used in previous studies.^14,15^ Participants with no data entry or reported missing data were classified as missing data.

Dementia was indicated by a clinical diagnosis based on UKB ICD9 or ICD10 diagnosis, read codes version 2 and/or self-reported dementia at all data collection time point. Dementia coding is available in UKB released algorithmically defined outcomes version 2 (https://biobank.ndph.ox.ac.uk/showcase/refer.cgi?id=460). Dementia cases were identified from primary care data using read code version 2 list in Wilkinson, Schnier.^16^ Participants who have reported any forms of dementia has been classified as ‘dementia cases’ and who have reported other diseases or no other disease were classified as ‘not having dementia’. Participants with no data entry or reported missing data were classified as missing data.

Out of the total 376,611 eligible participants classified as European and who passed the pre-GWAS sample QC (described in the next 2.3 GWAS section), there are 891 who have both periodontal diseases and dementia, 5133 dementia only, and 67,591 periodontal diseases only. There are 302,957 participants with data entry for dementia or periodontal diseases measurement (i.e., not reporting missing data) but without reporting either periodontal diseases or dementia considered as controls.

### Sample allocation

**Figure 1.**
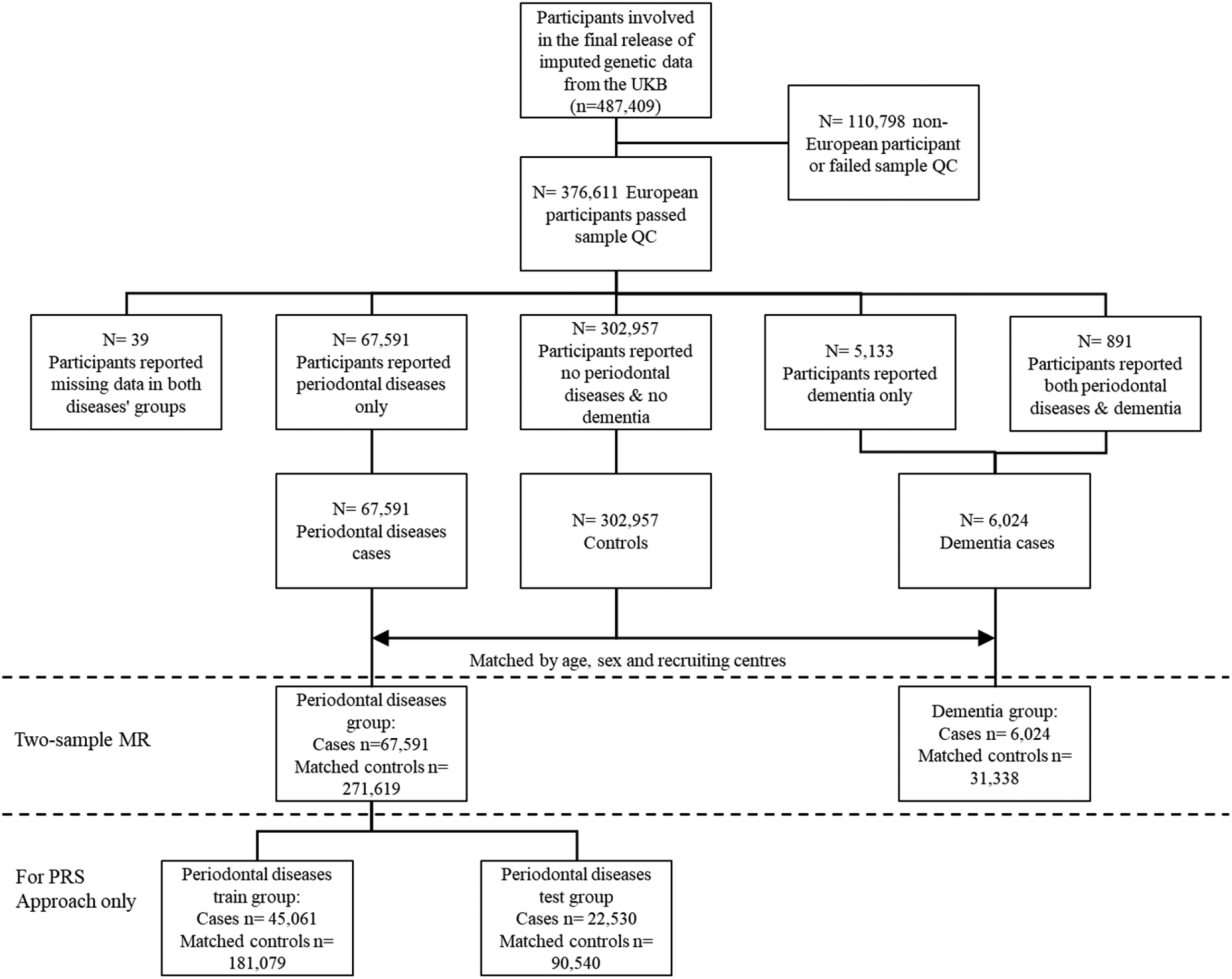
Sample allocation flow chart. UKB: UK Biobank; N, n: number of sample; QC: quality control; MR: Mendelian randomization; PRS: polygenic risk score.

First of all, participants with only periodontal diseases were allocated to the periodontal diseases group and those with only dementia to the dementia group, as two independent samples (Figure 1). Participants who reported both diseases were allocated to the dementia group to maximize the dementia group sample size. No participants were allocated into both sample groups to avoid sample overlap and subsequent weak instrument bias.^17^ Participants without periodontal diseases nor dementia (controls) were randomly allocated into the periodontal diseases or dementia group, matched by age, sex and recruiting center at baseline. The number of controls were more than three times of cases in each group to optimize statistical power.^18^

To facilitate the PRS analysis, the periodontal diseases sample were further allocated into a periodontal diseases “training” group and a periodontal diseases “test” group by randomly selecting cases and controls. The case-control ratio of 1:3 was retained in the periodontal diseases training and periodontal diseases test group (Figure 1).

### GWAS in UKB

UKB genotyped 487,409 participants using two genotyping arrays and performed imputation resulting in 93,095,623 autosomal variants by using UK10 K, 1000 Genomes phased and Haplotype Reference Consortium reference panels.^10^ We performed pre-GWAS QC on both sample and genetic variants in the European ancestry participants. The full details of GWASs performed in this study is available in the Supplemental Information. The GWAS was performed for periodontal diseases status in the periodontal diseases training group and periodontal diseases group, as well as for dementia status in dementia group by logistic regression model assuming an additive mode of inheritance. The GWAS models adjusted for covariates: age, sex, and the first 15 principal components (PCs) as provided by UKB.

### Two-sample MR and genetic instruments

Two-sample MR analysis were performed between the periodontal diseases group and the dementia group (traditional approach), as well as between the periodontal diseases train and the dementia group (PRS approach). Following GWA, clumping was performed to obtain independently significant single nucleotide polymorphisms (SNPs, threshold p-value < 10^−4^, linkage disequilibrium (LD) r^2^ < 0.1 and window size 250 kb). These SNPs were the potential genetic instruments. To further identify valid SNPs (genetic instruments), various selection approaches were utilized:

#### Main approach

Genetic instruments selection based on SNPs reaching conventional GWAS threshold (p < 5 × 10^−8^) from the periodontal diseases group.

#### Alternative approach I

Genetic instruments reaching suggestive level of significance (p < 5 × 10^−6^) within the periodontal diseases group.

#### Alternative approach II

SNPs that reached a suggestive level of significance (p < 10^−4^) were selected from the periodontal diseases group. This approach was utilized together with alternative approach I to cross-check differences with main approach and the robustness of any suggested causal relationship.

#### Alternative approach III

Genetic instruments reached the best-fit p-value threshold based on PRS were selected from periodontal diseases train group GWAS. Specifically, GWAS was performed in periodontal diseases train group, and then the PRS analysis was performed using GWAS summary statistics from periodontal diseases train group on periodontal diseases test group. The best-fit p-value threshold potentially provided better explanation of periodontal diseases variance in the sample which could again provide an extra sensitivity analysis to check the results consistency and robustness.

### Statistical analysis

#### MR

**Figure 2.**
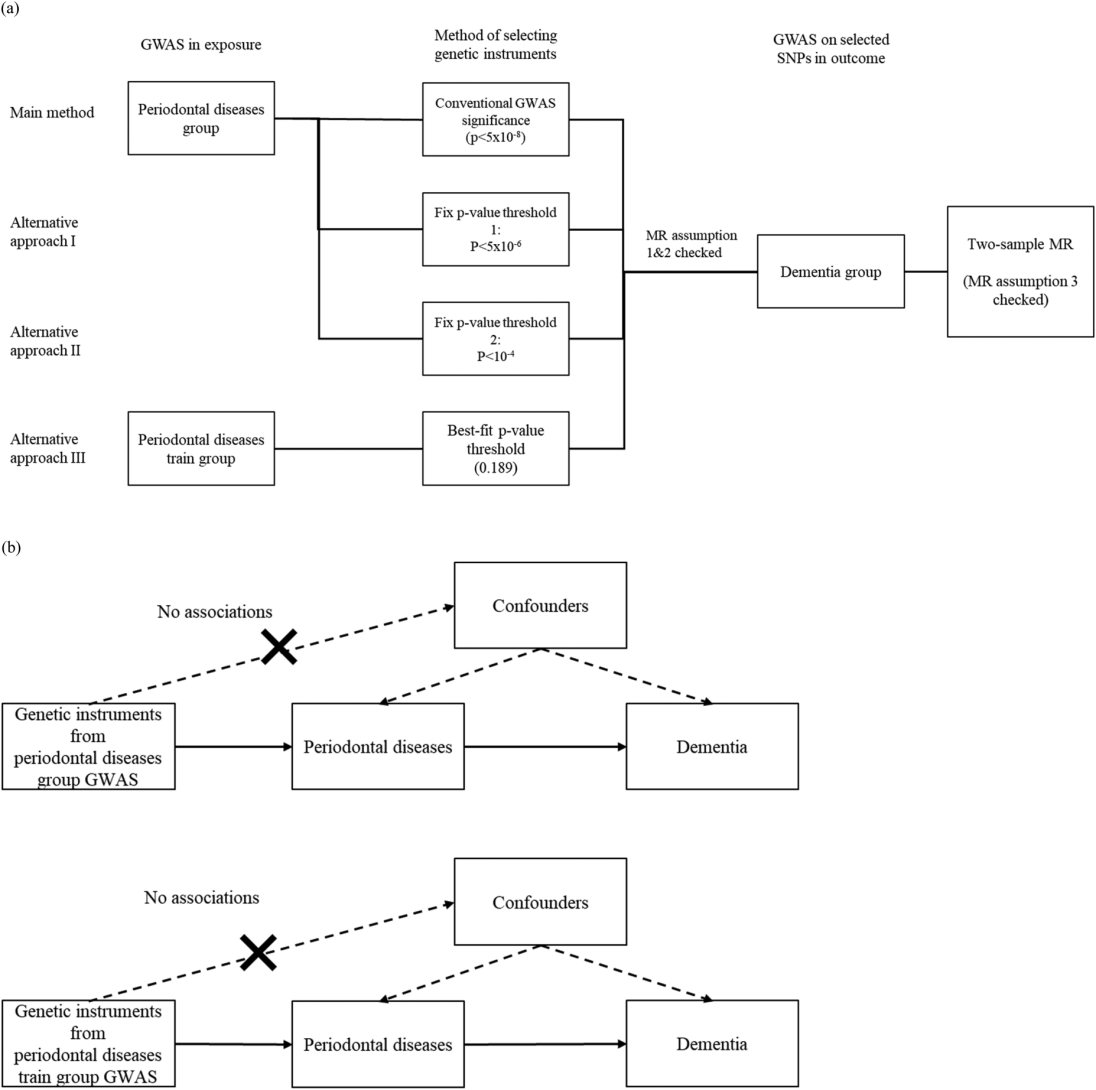
Three main MR analysis method in this study. (a) demonstrates the MR analysis procedures for all approaches utilized in current study from GWAS in periodontal diseases, genetic instruments selection, GWAS in dementia and finally two-sample MR. (b) MR models indicated in our study, the cross sign indicated there is no association allowed between genetic instruments and confounders.

Two-sample MR using these four models with four sets of genetic instruments were utilized in this study (Figure 2). Four MR methods were employed, including IVW MR,^17,19^ MR-Egger regression,^17,20,21^ Weighted median^17,22^ and mode-based estimate^23^ to test and cross validate the effect of periodontal diseases on dementia. These different methods reflect a variety of different assumptions and scenarios about the MR analysis.

The MR assumption check was performed prior to MR analysis. The relevance of genetic instrument (assumption 1) was ensured by selecting instrument using strict p-value thresholds (i.e., p < 5 × 10^−8^, p < 5 × 10^−6^, and p < 10^−4^; the best-fit p-value threshold obtained from PRS analysis) and F statistic of at least 10. The association with potential confounders (Assumption 2) were checked by regressing the selected instrument in each model on several confounders (socioeconomic status, smoking status, alcohol drinking status, blood level of C-reactive protein) with adjustment of age, sexm and PC1-15. Significant association with confounders after adjusting for multiple testing using Bonferroni correction indicates violation of assumption 2 and the SNP(s) were removed from further analysis. The horizontal pleiotropy (Assumption 3) was assessed by the MR-Egger regression intercept.

In addition to methods above, MR on each individual SNPs was performed for strongest genetic instruments (selected based on p < 5 × 10^−8^) to gain better insight on their effects in the association.

The GWA and clumping were implemented in PLINK 2.0 (Linux) (https://www.cog-genomics.org/plink/2.0/).^24^ Manhattan plots and Q-Q plots were created in R 4.4.2 (https://www.r-project.org/about.html), using the packages “qqman”^25^ and “fastqq” (https://github.com/gumeo/fastqq). The PRS analysis weas performed using PRSice2 (https://choishingwan.github.io/PRSice/).^26^ The MR analysis was conducted in R 4.4.2 using package “TwoSampleMR”^27,28^ and “MendelianRandomization”^29,30^

## Results

### Sample characteristics

Sample QC included: excluding 378 participants with sex mismatch between biological sex and self-reported sex, removing 968 participants with high SNP missing rates (>5%) and unusually high heterozygosity rate as documented by UKB, plus removing 40,196 related participants (kinship coefficient > 0.08838835^31^) where one participant was dropped randomly from every related pair. The QC on genetic variants also excluded the SNPs with minor allele frequency < 0.001 and imputation information score ≤ 0.5, resulting in 18,849,429 SNPs from the autosomal chromosomes included in association analysis. As a result of this QC, 376, 611 European participants from UKB passed sample QC.

Further 39 UKB participants were removed from the analysis due to missing data in both periodontal diseases and dementia measurements. Among the remaining 376,572 participants (Mean age 70.45, Standard Deviation 7.89), 18.2% participants had periodontal diseases and 1.6% of them were diagnosed or self-reported with all-causes dementia. Sex was balanced in the remaining sample with slightly more female present (53.8%). Then participants were allocated into dementia and periodontal diseases groups. The dementia group has higher mean age (dementia group 76.54 (SD 5.3) vs. periodontal diseases group 69.78 (SD 7.84)) and has fewer female participants (46.8% dementia group vs. 54.5% periodontal diseases group). There were 891 participants (14.9% of dementia cases) experienced both periodontal diseases and dementia and they have been allocated to dementia group. Sample characteristics for each group were described in Table 1. Sample characteristics of Periodontal diseases Group and periodontal diseases train group including selected confounders’ summary statistics were included in Supplemental Table 1.

**Table 1.** Sample characteristics.

	Overall	Periodontal diseases group		Dementia group	
		Cases	Controls	Cases	Controls
N	376,572	67,591	271,619	6024	31,338
Age (Mean (SD))	70.45 (7.89)	69.11 (7.75)	69.95 (7.85)	76.48 (5.30)	76.55 (5.30)
Sex = Female (%)	202,440 (53.8)	40,382 (59.7)	144,554 (53.2)	2843 (47.2)	14,661 (46.8)
Periodontal Diseases = Yes (%)	68,482 (18.2)			891 (14.9)	0 (0.0)
Dementia = Yes (%)	6024 (1.6)	0 (0)	0 (0)		

SD: standard deviation.

### GWAS results and clumping

To enable downstream MR analysis, GWAS were performed in the periodontal diseases group and the periodontal diseases train group.

In the periodontal diseases group, five SNPs reached conventional GWAS significance (p < 5 × 10^−8^): rs368467810[TTTA], rs12123266[G], rs28381639[G], rs12124244[G], rs6696651[A]. There were 571 SNPs reach p < 5 × 10^−6^ significance level, and 5053 SNPs reached p < 10^−4^ level. Clumping resulted in 1020 leading SNPs at suggestive level (maximum p = 10^−4^) and three SNPs at conventional significant level p < 5 × 10^−8^ (Supplemental Table 2). The Manhattan plot and Q-Q plot can be viewed in Supplemental Figure 1. In the periodontal diseases train group GWAS (Supplemental Figure 2), 3767 SNPs reached p < 10^−4^ level, and 970 independent significant SNPs were obtained after clumping (Supplemental Table 3). The F-statistic in all MR approaches > 10 suggesting sufficient strength of genetic instruments (assumption 1).

Association between the confounders and the independent SNPs (i.e., potential genetic instruments) revealed 27 SNPs from periodontal diseases group GWAS and 12 SNPs from periodontal diseases train group GWAS violated assumption 2 and have been removed from further analysis.

### Main approach (significance level p < 5 × 10^−8^)

The main approach MR was performed on two genome-wide significant SNPs (n = 2) (i.e., rs368467810, rs12123266) (F statistics = 32.2, R^2^ = 0.02%) which passed the assumption 2 check from the periodontal diseases group GWA study, showing weak association (Beta: −0.816, 95%CI (−1.617, −0.015), p = 0.046) in the IVW model with no evidence of heterogeneity (Cochran's Q = 0.32 on 1 degrees of freedom (df), p = 0.57) (Table 2). Results from the mode-based estimate were marginal (p = 0.051), while MR Egger regression and weighted median analysis were not performed due to insufficient number of genetic instruments. Due to the inconsistent result between models, we further performed MR on each individual SNPs. The results showed that no association between periodontal diseases and dementia by using single SNPs.

**Table 2.** MR results from all models.

	**β (95%CI)**	**p**	**Cochran's Q (df)**	**p_heterogeneity_**
**Main Approach: SNPs (p** **<** **5** **×** **10^−8^) From Periodontal Diseases Group (SNPs N** **=** **2, F** **=** **32.2, R^2^** **=** **0.02%)**				
MR IVW	−0.816 (−1.617, −0.015)	0.046*	0.32 (1)	0.57
Mode-Based Estimate	−0.820 (−1.643, 0.003)	0.051		
**Alternative Approach 1: SNPs (p** **<** **5** **×** **10^−6^) From Periodontal Diseases Group (SNPs N** **=** **79, F** **=** **23.2, R^2^** **=** **0.54%)**				
MR IVW	−0.070 (−0.222, 0.083)	0.37	76.73 (78)	0.52
MR-Egger Regression	0.048 (−0.197, 0.294)	0.699		
MR-Egger Regression Intercept	−0.008 (−0.020, 0.005)	0.229		
MR Weighted Median	−0.066 (−0.282, 0.150)	0.548		
Mode-Based Estimate	−0.348 (−0.932, 0.237)	0.243		
**Alternative Approach 2: SNPs (p** **<** **10^−4^) From Periodontal Diseases Group (SNPs N** **=** **993, F** **=** **17.4, R^2^** **=** **6.40%)**				
MR IVW	0.025 (−0.024, 0.075)	0.316	954.00 (992)	0.8
MR-Egger Regression	−0.008 (−0.080, 0.063)	0.82		
MR-Egger Regression Intercept	0.003 (−0.001, 0.006)	0.197		
MR Weighted Median	0.077 (0.006, 0.149)	0.035*		
Mode-Based Estimate	0.235 (−0.090, 0.560)	0.157		
**Alternative Approach 3: SNPs (p** **<** **0.19) From Periodontal Diseases Train Group (SNPs N** **=** **958, F** **=** **17.4, R^2^** **=** **12.39%)**				
MR IVW	0.035 (−0.007, 0.076)	0.101	848.24 (957)	0.99
MR-Egger Regression	0.044 (−0.015, 0.103)	0.147		
MR-Egger Regression Intercept	−0.001 (−0.005, 0.003)	0.677		
MR Weighted Median	0.035 (−0.025, 0.095)	0.25		
Mode-Based Estimate	0.039 (−0.231, 0.308)	0.779		
**MR: rs12123266 (p** **<** **5** **×** **10^−8^) From Periodontal Diseases Group (F** **=** **31.3, R^2^** **=** **0.01%)**				
MR IVW	−0.577 (−1.734, 0.581)	0.329		
**MR: rs368467810 (p** **<** **5** **×** **10^−8^) From Periodontal Diseases Group (F** **=** **33.2, R^2^** **=** **0.01%)**				
MR IVW	−1.036 (−2.145, 0.073)	0.067		

β: coefficient beta; p: p-value; CI: confidence interval; df: degree of freedom; SNP: single nucleotide polymorphism; MR: Mendelian randomization; IVW: inverse-variance weighted.

### Alternative approach I and II (significance level at p < 5 × 10^−6^ and 10^−4^)

When lifting the genetic instrument selection criteria to p < 5 × 10^−6^ (SNPs n = 79) (F = 23.2, R^2^ = 0.54%), IVW model suggested no association between periodontal diseases and dementia (Beta: −0.070, 95%CI(−0.222, 0.083), p = 0.37, neither in other MR methods. There is no evidence of horizontal pleiotropy and heterogeneity indicated by MR-Egger regression intercept (p = 0.229) and heterogeneity test (Cochran's Q = 76.73 on df 78, p = 0.52) (Table 2).

In the alternative approach 2 (p < 10^−4^) (F = 17.4, R^2^ = 6.40%), there were 993 SNPs included in the MR analysis. No associations were found in MR IVW (Beta: 0.025, 95%CI(−0.024, 0.075), p = 0.316), nor in MR-Egger regression and mode-based estimate model. However, MR weighted-median model showed a weak positive causal relationship between periodontal diseases and dementia (Beta: 0.077, 95%CI (0.006, 0.149), p = 0.035). There was also no sign of heterogeneity (Cochran's Q = 954.00 on 992 df, p = 0.8) and horizontal pleiotropy (MR-Egger regression intercept p = 0.197).

### Alternative approach III using PRS approach

As PRS results on periodontal diseases test group showed best-fit p-value threshold is 0.19, all leading SNPs in periodontal diseases train group after clumping has been included in the MR analysis (n = 958, F = 17.4, R^2^ = 12.39%). The IVW (Beta: 0.035 95%CI(−0.007, 0.076), p = 0.101) and all other models based on SNPs from the periodontal diseases training sample were insignificant suggested no evidence for causal relationship between periodontal diseases and dementia. Again, there is no evidence of heterogeneity (Cochran's Q = 848.24 on 957 df, p = 0.99) and horizontal pleiotropy (MR-Egger regression intercept p = 0.677) (Table 2).

## Discussion

Using multiple approaches to identify valid genetic instruments for periodontal diseases, we found no convincing evidence to support a causal relationship between periodontal diseases and dementia in UKB participants of European ancestry. Although weak associations emerged in some models, these are likely spurious findings, given the limitations of self-reported periodontal diseases measures.

Using epidemiological approach, many studies suggested periodontal diseases is associated with a higher risk of dementia.^2,32,33^ However, due to the heterogeneity of study design factors (ethnicity, periodontal diseases measurements and definition, sample size), their association is not conclusive.^3^ Shared causes instead of causal relationship may be one of the possible explanations for missing causation in the MR studies. Chronic inflammatory diseases are polygenic involving genes functioning within the immune response being shared between various inflammatory diseases^34^ where both periodontal diseases and dementia is characterized by inflammation^35,36^ A meta-analysis of population study has shown that higher levels of systemic inflammatory markers, including C-reactive protein and interleukin (IL)-6, are associated with increased dementia risk,^37^ while systemic inflammation may promote neurodegeneration through transport of proinflammatory cytokines to the brain crossing blood-brain barrier, and producing proinflammatory microglial and astrocytic phenotypes, which may contribute to tau hyperphosphorylation, amyloid-β oligomerization, complement activation, and the breakdown of neurotransmitters into potentially harmful bioactive metabolites.^38^ Periodontal disease has been associated with elevated circulating inflammatory mediators, including IL-1, IL-6, C-reactive protein, and fibrinogen, as well as decreased markers level following periodontal treatments.^39–42^ Recent genetic study also suggests a close link between periodontal diseases and Alzheimer's disease pathogenesis with shared inflammation-related genes and pathways, including IL-6, IL-1β, IL-10, and C-reactive protein.^43^ Although in our study we excluded genetic instruments associated with blood levels of C-reactive protein, some other unmanaged shared inflammatory related gene may pose risk of undetected horizontal pleiotropy in MR analyses. This highlights the importance of cautious instrument selection and suggests that future MR studies using genetic instruments for systemic inflammatory markers, such as IL-6 or C-reactive protein, may help clarify whether inflammation plays a more direct causal role in dementia than periodontal phenotypes alone.

In addition to the inflammatory responses, many modifiable environmental factors, such as smoking and nutrition intake, as important causal risk factors for both periodontal diseases and dementia that are not well adjusted for in all epidemiological studies.^44^ Since both periodontal diseases and dementia are all multi-factorial diseases with various factors play important role in the disease's progression, missing causality may be due to shared confounders. Especially, periodontal diseases have only low to moderate heritability where the role of environmental factors either acting solely or interactively with genetic risk factors in periodontal diseases may be more representative to periodontal diseases progression. This would lead to the difficulty of selecting robust genetic instruments and find a causal conclusion between periodontal diseases and dementia. For example, in our MR models, the variance of periodontal diseases explained by the genetic instruments selected ranged from (R^2^ 0.01%–12.39%) which potentially decrease the precision of MR estimates^45^ and may highlight multifactorial nature of periodontal diseases. The reverse causation, which means dementia increases the risk of periodontal diseases, is another possibility for not observing causation in the direction of periodontal diseases affecting dementia but commonly reported in observational studies. Life Course hypothesis suggested that poor cognitive function in childhood contributes to a cumulative effect throughout the life span (i.e., poorer academic achievement and education level, poorer oral hygiene, lifestyle behaviors including alcohol drinking and smoking) which finally led to incremental effect on tooth loss, the final stage of periodontal diseases, at older age and poor cognitive outcome.^44^ Therefore, further investigation on the mechanisms underlying the association between periodontal diseases and dementia observed in epidemiology study is still needed.

On the other hand, unlike many diseases such as cancer, where consensus of genes that are truly associated with the risk of disease is confirmed and supported by different source of evidence, the development of GWAS in periodontal diseases still has a long way to go. The inconsistency in GWAS results in periodontal diseases, both in our study and others, indicates the challenges in selecting genetic instruments for periodontal diseases using MR approach to understand the causation between periodontal diseases and any systemic diseases, including dementia.

There are several strengths of the current study. First, we utilized various methods to validate the casual association between periodontal diseases and dementia by using large scale data from UKB. Secondly, instead of using one-sample MR directly on UKB, our study aplite the sample into two to the apply the two-sample MR method. This not only minimized the confounding effect and reverse causation but also reduce the bias due to overfitting which often experienced in one-sample MR.^17^ Thirdly, by applying two-sample MR in a single largescale dataset, the power and similarities between samples were ensured. The limitation in this study is mainly the self-reported measurement used for defining periodontal diseases. Though this symptom-based measurement is a valid periodontal diseases measurement in absence of clinical examination for periodontal diseases classification,^12,13^ potential risk of misclassification (e.g., other diseases with overlapping symptoms) may potentially bias the results, so interpretation with cautious is needed. Clinical examination on periodontal status using the 2018 periodontal status classification criteria^46^ is recommended in future study. Another limitation is the small number of dementia cases (N = 6024), as the UKB is composed of relative healthier population than general public and the dementia cases can be much underestimated.

### Conclusion

We did not find consistent causal relationship between periodontal diseases and dementia using various models and selection methods of genetic variants of periodontal diseases. Validated genetic instruments of periodontal diseases are needed to ensure MR approach is appropriate to use in future studies.

## Supplemental Material

sj-docx-1-alz-10.1177_13872877261450558 - Supplemental material for Periodontal diseases and all-cause dementia risk: Genetic instrument analyses in half a million UK Biobank participants Supplemental material, sj-docx-1-alz-10.1177_13872877261450558 for Periodontal diseases and all-cause dementia risk: Genetic instrument analyses in half a million UK Biobank participants by Chenyi Gao, Mark M. Iles, David Bunce, Bei Wu, Huabin Luo, Susan Pavitt, Jianhua Wu, David Timothy Bishop and Jing Kang in Journal of Alzheimer's Disease

sj-xlsx-2-alz-10.1177_13872877261450558 - Supplemental material for Periodontal diseases and all-cause dementia risk: Genetic instrument analyses in half a million UK Biobank participants Supplemental material, sj-xlsx-2-alz-10.1177_13872877261450558 for Periodontal diseases and all-cause dementia risk: Genetic instrument analyses in half a million UK Biobank participants by Chenyi Gao, Mark M. Iles, David Bunce, Bei Wu, Huabin Luo, Susan Pavitt, Jianhua Wu, David Timothy Bishop and Jing Kang in Journal of Alzheimer's Disease

sj-xlsx-3-alz-10.1177_13872877261450558 - Supplemental material for Periodontal diseases and all-cause dementia risk: Genetic instrument analyses in half a million UK Biobank participants Supplemental material, sj-xlsx-3-alz-10.1177_13872877261450558 for Periodontal diseases and all-cause dementia risk: Genetic instrument analyses in half a million UK Biobank participants by Chenyi Gao, Mark M. Iles, David Bunce, Bei Wu, Huabin Luo, Susan Pavitt, Jianhua Wu, David Timothy Bishop and Jing Kang in Journal of Alzheimer's Disease
