# Supplementary material for: Periodontal diseases and all-cause dementia risk: Genetic instrument analyses in half a million UK Biobank participants
Source: J Alzheimers Dis. 2026 May 21;112(1):273–82. doi: 10.1177/13872877261450558 (PMC13291395; doi:10.1177/13872877261450558)
Supplement: sj-docx-1-alz-10.1177_13872877261450558 - Supplemental material for Periodontal diseases and all-cause dementia risk: Genetic instrument analyses in half a million UK Biobank participants [file sj-docx-1-alz-10.1177_13872877261450558.docx]

**Supplemental Material**

**Periodontal diseases and all-cause dementia risk: Genetic instrument analyses in half a million UK Biobank participants**

**Supplemental Information**

*Genome-wide association analysis (GWA)*

The GWA was performed for periodontal diseases status in the periodontal diseases training group and periodontal diseases group, as well as for dementia status in dementia group. The aim of conducting GWA in periodontal diseases group and periodontal diseases train group separately is to facilitate the two distinct genetic instruments selection methods: (1) use of the traditional fixed p-value threshold to select the genetic instrument in the periodontal diseases group; (2) use of the best-fit p-value threshold computed by polygenic risk score (PRS) analysis in the periodontal diseases training and test group. A logistic regression model assuming an additive mode of inheritance was applied across all GWA performed, as well as with adjustment for covariates: age, sex, and the first 15 principal components as provided by UKB.

The GWA results of periodontal diseases from the periodontal diseases train group were further used to estimate how well these SNPs can predict periodontal diseases in periodontal diseases test group and estimating the best fit p-value threshold for the PRS approach.

**Supplemental Figure 1.** Manhattan plot and QQ plot of periodontal diseases group GWAS.

**(a) (b)**


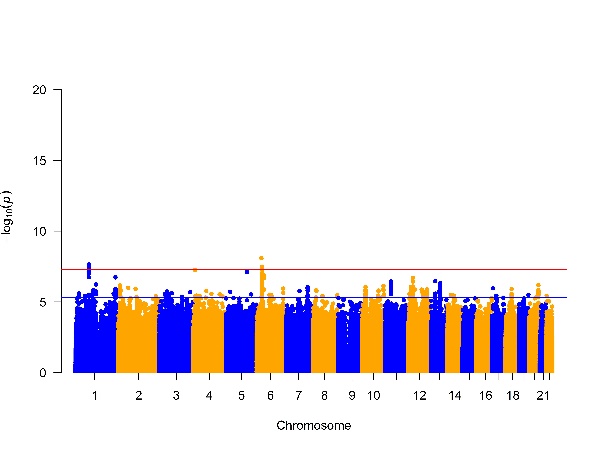

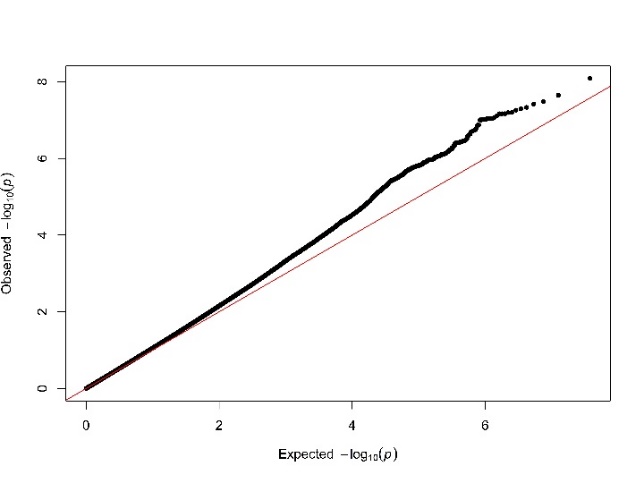


(a) Manhattan plot of periodontal diseases GWAS in periodontal diseases group. The red line indicated the conventional GWAS significant level (p<5e-8). The blue line indicates the suggestive significant level (p<1e-5). (b) Q-Q plot of periodontal diseases GWAS in periodontal diseases group (genomic control inflation factor lambda = 1.07). The red line represents the reference line.

**Supplemental Figure 2**. Manhattan plot and Q-Q plot of periodontal diseases train group GWAS.

**(a) (b)**


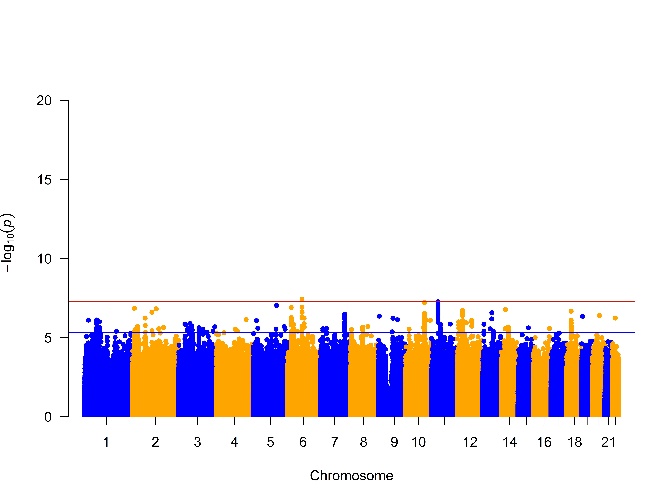

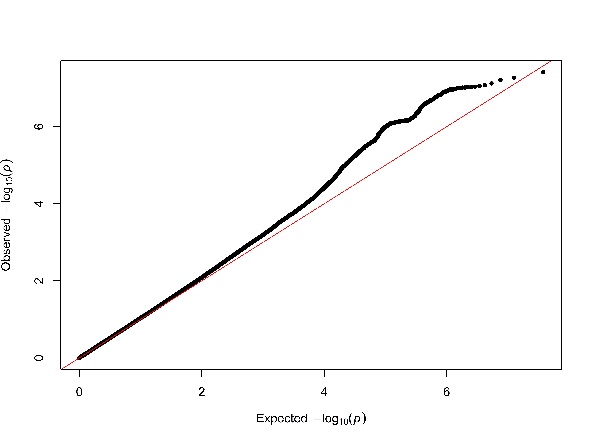


(a) Manhattan plot of periodontal diseases GWAS in periodontal diseases train group. The red line indicated the conventional GWAS significant level (p<5e-8). The blue line indicates the suggestive significant level (p<5e-6). (b) Q-Q plot of periodontal diseases GWAS in periodontal diseases train group (genomic control inflation factor lambda = 1.05). The red line represents the reference line.

**Supplemental Table 1.** Sample characteristics for periodontal diseases and periodontal diseases train group participants.

|  | **Periodontal diseases Group** | | **Periodontal diseases Train Group** | | |  |
| --- | --- | --- | --- | --- | --- | --- |
|  | **Case** | **Control** | | **Case** | **Control** | |
| N | 271619 | 67591 | | 181079 | 45061 | |
| Age (Mean (SD)) | 69.95 (7.85) | 69.11 (7.75) | | 69.68 (7.84) | 69.08 (7.75) | |
| Sex = Female (%) | 144554 (53.2) | 40382 (59.7) | | 99714 (55.1) | 26934 (59.8) | |
| Periodontal diseases = Yes (%) | 0 (0.0) | 67591 (100.0) | | 0 (0.0) | 45061 (100.0) | |
| Dementia = Yes (%) | 263906 (100.0) | 66062 (100.0) | | 175797 (100.0) | 44052 (100.0) | |
| Household Income (%) |  |  | |  |  | |
| Less Than 18,000 | 48318 (20.6) | 13326 (22.5) | | 31891 (20.4) | 8852 (22.5) | |
| 18,000 To 30,999 | 59151 (25.2) | 15001 (25.4) | | 39196 (25.0) | 10037 (25.5) | |
| 31,000 To 51,999 | 63129 (26.9) | 15952 (27.0) | | 42434 (27.1) | 10567 (26.8) | |
| 52,000 To 100,000 | 50561 (21.5) | 12152 (20.6) | | 33955 (21.7) | 8143 (20.7) | |
| Greater Than 100,000 | 13532 (5.8) | 2678 (4.5) | | 9156 (5.8) | 1806 (4.6) | |
| Smoking Status |  |  | |  |  | |
| Never | 151466 (56.0) | 34825 (51.7) | | 101509 (56.2) | 23240 (51.7) | |
| Ex-Smoker | 91685 (33.9) | 25602 (38.0) | | 60534 (33.5) | 17034 (37.9) | |
| Current Smoker | 27551 (10.2) | 6960 (10.3) | | 18440 (10.2) | 4656 (10.4) | |
| Alcohol Status |  |  | |  |  | |
| Never | 8306 (3.1) | 1764 (2.6) | | 5582 (3.1) | 1184 (2.6) | |
| Former Drinker | 8929 (3.3) | 2541 (3.8) | | 5958 (3.3) | 1671 (3.7) | |
| Current Drinker | 254165 (93.6) | 63229 (93.6) | | 169392 (93.6) | 42166 (93.7) | |
| BMI (Mean (SD)) | 27.30 (4.70) | 27.82 (5.07) | | 27.26 (4.72) | 27.82 (5.10) | |
| C-Reactive Protein Level (Mean (SD)) | 2.09 (2.37) | 2.29 (2.52) | | 2.09 (2.38) | 2.29 (2.51) | |

SD: standard deviation; BMI: body mass index

**Supplemental Table 2.** Genetic instruments involved in MR analysis from periodontal diseases GWAS (periodontal diseases group) and corresponding GWAS results from dementia GWAS (dementia group).

*(Please see Excel file “SupplementalTable2.xlsx”)*

CHROM: chromosome; POS: position; SNP: single nucleotide polymorphism; A1: effect allele; A2: non-effect allele; EAF: effect allele frequency; BETA: coefficient beta; SE: standard error; p: p-value. Note. Exposure here refers to periodontal diseases and outcome refers to dementia. The SNPs reached conventional GWAS significant level were highlighted.

**Supplemental Table 3.** Genetic instruments involved in MR analysis from periodontal diseases GWAS (periodontal diseases train group) and corresponding GWAS results from dementia GWAS (dementia group).

(Please see Excel file “SupplementalTable3.xlsx”)

CHROM: chromosome; POS: position; SNP: single nucleotide polymorphism; A1: effect allele; A2: non-effect allele; EAF: effect allele, frequency; BETA: coefficient beta; SE: standard error; p: p-value; F_stats: F-statistics. Note. Exposure here refers to periodontal diseases and outcome refers to dementia.
